# Prevalence of comorbidity in Chinese patients with COVID-19: systematic review and meta-analysis of risk factors

**DOI:** 10.1186/s12879-021-05915-0

**Published:** 2021-02-22

**Authors:** Tingxuan Yin, Yuanjun Li, Ying Ying, Zhijun Luo

**Affiliations:** 1 Queen Mary School, Nanchang University, Nanchang, Jiangxi China; 2 Jiangxi Province Key Laboratory of Tumor Pathogenesis and Molecular Pathology and Department of Pathophysiology, School of Basic Medical Sciences, Nanchang University, Nanchang, Jiangxi China

**Keywords:** COVID-19, Comorbidity, Mortality, Meta-analysis, Risk factors

## Abstract

**Background:**

Coronavirus disease 2019 (COVID-19) is an infectious disease characterized by cough, fever, and fatigue and 20% of cases will develop into severe conditions resulting from acute lung injury with the manifestation of the acute respiratory distress syndrome (ARDS) that accounts for more than 50% of mortality. Currently, it has been reported that some comorbidities are linked with an increased rate of severity and mortality among COVID-19 patients. To assess the role of comorbidity in COVID-19 progression, we performed a systematic review with a meta-analysis on the relationship of COVID-19 severity with 8 different underlying diseases.

**Methods:**

PubMed, Web of Science, and CNKI were searched for articles investigating the prevalence of comorbidities in severe and non-severe COVID-19 patients. A total of 41 studies comprising 12,526 patients were included.

**Results:**

Prevalence of some commodities was lower than that in general population such as hypertension (19% vs 23.2%), diabetes (9% vs 10.9%), chronic kidney disease (CKD) (2% vs 9.5%), chronic liver diseases (CLD) (3% vs 24.8%) and chronic obstructive pulmonary disease (COPD) (3% vs 8.6%), while some others including cancer (1% vs 0.6%), cardiovascular disease (6% vs 1.8%) and cerebrovascular disease (2% vs 0.9%) exhibited greater percentage in COVID-19. Cerebrovascular disease (OR = 3.70, 95%CI 2.51–5.45) was found to be the strongest risk factor in disease exacerbation, followed by CKD (OR = 3.60, 95%CI 2.18–5.94), COPD (OR = 3.14, 95% CI 2.35–4.19), cardiovascular disease (OR = 2.76, 95% CI 2.18–3.49), malignancy (OR = 2.63, 95% CI 1.75–3.95), diabetes (OR = 2.49, 95% CI 2.10–2.96) and hypertension (OR = 2.13, 95% CI 1.81–2.51). We found no correlation between CLD and increased disease severity (OR = 1.32, 95% CI 0.96–1.82).

**Conclusion:**

The impact of all eight underlying diseases on COVID-19 deterioration seemed to be higher in patients outside Hubei. Based on different comorbidities, COVID-19 patients tend to be at risk of developing poor outcomes to a varying degree. Thus, tailored infection prevention and monitoring and treatment strategies targeting these high-risk subgroups might improve prognosis during the COVID-19 pandemic.

**Supplementary Information:**

The online version contains supplementary material available at 10.1186/s12879-021-05915-0.

## Background

From December of 2019 to March of 2020, an outbreak of pneumonia caused by severe acute respiratory syndrome coronavirus-2 (SARS-CoV-2), a novel type of coronavirus sharing 79.5% genome identity with SARS-CoV, was first found in Wuhan, Hubei province, and spread rapidly to other cities and provinces across China. The World Health Organization (WHO) has officially named this coronavirus as the 2019 novel coronavirus (2019-nCoV) and the corresponding disease as COVID-19 [1]. Compared to previously described acute respiratory infectious diseases, SARS in 2003 and the Middle East Respiratory Syndrome (MERS) in 2012, COVID-19 shows lower mortality ranging from 0.7 to 4%, but enhanced transmission due to more than ten times higher affinity to common target ACE2, causing a rapid spread worldwide [2]. COVID-19 patients manifest different degrees of clinical symptoms as mild, moderate, severe, and critical illness. Many patients with COVID-19 initially present flu-like symptoms as fever (91.3%), dry cough (67.7%), and fatigue (51.0%), followed by dyspnea (30.4%) [3]. Most patients with these symptoms have a good prognosis, while only a small portion of patients will convert into severe or critical cases, rapidly developing lethal complications (such as acute respiratory distress syndrome, septic shock, and irreversible metabolic acidosis) and even death, especially for the elderly and those with underlying diseases [4]. Moreover, it is found that many comorbidities correlate with the severity of COVID-19. Thus, it is critical to identify the sub-population that is more susceptible to the development of adverse outcomes of COVID-19 and prevent the deterioration from mild and moderate conditions to the severe ones and reduce mortality. Furthermore, the assessment of the specific risk factor underlying different comorbidities is conducive to the special care of the targeted population.

Thus far, common concerns presented from recent studies regarding the prevalence of comorbidity in severe COVID-19 patients include limited sample size, isolated data of the one-center study, and inconsistent conclusions, making it difficult to have an overall awareness. Thus, it is necessary to carry out a meta-analysis to evaluate convincing outcomes of this issue. This study aims to provide systematic evaluation and detailed estimation of prevalence rates of different kinds of common comorbidities in severe and non-severe COVID-19 patients and tries to illustrate the outcomes by combination with underlying pathogenesis to better understand how basic diseases contribute to the aggravation of SARS-CoV-2 infection.

## Method

The protocol of this systematic review and meta-analysis was registered at the International Prospective Register of Systematic Reviews (PROSPERO) and the registration number is CRD42020178826. This meta-analysis was performed under the guidance of the Preferred Reporting Items for Systematic Reviews and Meta-Analysis (PRISMA) statement (An additional file shows this following the checklist in more detail [see Additional file 1]).

### Search strategy

To identify all the studies illustrating the prevalence of comorbidities of SARS-CoV-2 infection in China, the international databases, including PubMed and Web of Science, and the Chinese database CNKI were searched for articles published till 18 January 2021. The search items and corresponding synonyms were combined with Boolean operators “AND” and “OR” as follows:

“COVID-19” or “2019-nCoV” or “novel coronavirus” or “SARS-CoV-2” or “Coronavirus”.
“comorbidities” or “characteristics” or “clinical features” or “underlying diseases” or “basic diseases” or “condition”.
“China” or “Chinese”.
(1) AND (2) AND (3)

#### Inclusion criteria

(1) Designed studies published online. (2) Studies contained well-recorded clinical characteristics and epidemiological information of patients diagnosed with COVID-19, including age, gender, symptoms, date of hospitalization, and comorbidities. (3) Participants in the study must be stratified into different groups according to severity or disease progression, in the form of either mild/moderate/severe/critical illness based on clinical symptoms or ICU/non-ICU based on admission care. Additionally, the basis of classification should be clearly and officially defined.

#### Exclusion criteria

(1) Non-human studies, case report, systematic reviews or abstract only; (2) Those with unavailable data or incomplete information collected from study subjects; (3) Repeated studies; (4) Pre-print articles; (5) Cases from foreign countries outside China; (6) Cases merely classified on survival and death.

### Literature selection and data extraction

Two reviewers (Y.T. and L.Y.) searched and selected the studies individually. First, titles and abstracts were screened. Second, full-text articles of potential interest were screened to decide whether the literature should be included according to the inclusion and exclusion criteria. The information extracted from the literature includes authors, duration of clinical observation, the specific hospital that cases come from, sample size, gender, age, respective numbers of severe and non-severe cases with and without any comorbidity, and the proportion of 4 common clinical symptoms among patients, including fever, cough, myalgia and dyspnea. The above information of included articles was encoded into an Excel Spreadsheets according to different basic diseases. (An additional file shows the data in more detail [see Additional file 2]) Then the numbers of specific comorbidities appearing in severe and non-severe patients, including hypertension, cardiovascular disease, diabetes, COPD, malignancy, CKD, CLD, and cerebrovascular disease, were extracted from the identified studies.

The primary outcome of this systematic review and meta-analysis was the prevalence of comorbidities among all included COVID-19 patients no matter the severity and was calculated as the proportion of those with one of 8 widespread basic diseases among all confirmed anticipants. The secondary outcome was identifying and estimating the risk comorbidities posed on the progression of COVID-19. The measure of the secondary outcomes was to compare the prevalence of 8 types of specific comorbidities in severe and non-severe cases (mild and moderate clinical types were regarded as non-severe, while severe and critically-ill were considered as severe). If patients were not divided into four standard clinical types, then we extracted the ICU and non-ICU cases as severe and non-severe instead, respectively. For cohort studies, after short-term following up, regarded those experiencing disease progression or poorer outcomes as severe, and those still in normal condition or better outcomes as non-severe.

### Data analysis and assessment of risk bias

All data analyses were performed by STATA 15, and article deduplication was done with Note-Express. Forest plots were used to visually illustrate the distribution of the outcome and effect size obtained from each included study, and to demonstrate the pooled prevalence and the effect on the severity of respective comorbidities in COVID-19 from the selected studies. The pooled prevalence and 95% confidence intervals (CI) were calculated for each of the selected comorbidities. The odd risk (OR) and 95% CI were adopted to describe the effect size of different underlying diseases on the development of severe cases. Statistical significance of the difference (the *P*-value of Z zone from overall test effect) was set as *P* < 0.05 [5]. Statistical heterogeneity within and between studies was evaluated by Cochrane’s Q test for presence and I^2^ statistics for extent. To avoid false-positive results, the heterogeneity was indicated to be significant when *P* ≤ 0.1 or I^2^ ≥ 50% was performed [6]. Random-effects model was applied to all analyses, regardless of heterogeneity.

To preliminarily assess whether these basic diseases increase the susceptibility to SARS-CoV-2 infection, we compared the prevalence of these comorbidities between COVID-19 patients and overall Chinese population by u-test which is intended to infer whether the unknown total rate π represented by the sample is different from the known total rate π0. Generally, when np ≥ 5 and n (1-p) ≥ 5 (n: the sample size, p: the sample rate), the distribution of the sample rate is similar to normal distribution, and the hypothesis test of the difference between the sample rate and the overall population rate can be tested by using the principle of normal distribution. In this analysis, π_0_ was extracted from the latest published authoritative data of the prevalence of different comorbidities across China, p was obtained from the result of this meta-analysis on prevalence, and n refers to the number of included cases as 12,526. Set α = 0.05 as threshold, if π > 1.96, the difference between pooled prevalence from meta-analysis and the overall prevalence in China is statistically significant and thus not caused by sampling error but COVID-19. (The detailed computational procedures and formulas of u-test are available in additional file [see Additional file 2]).

To further test the correlation of comorbidity with adverse outcomes and assess whether the impact of infected areas was associated with comorbidities to disease progression, subgroup analysis based on patient source was conducted. Studies were stratified into two subgroups according to the region of patient source, one group contained cases only from Hubei province, and the other focused on regions out of Hubei. Besides, subgroup analysis was used to test sensitivity by exploring potential sources of heterogeneity from any of the two subgroups. The risk of publication bias was assessed by Begg rank correlation test and Egger linear regression test, and *P* < 0.05 indicated that significant publication bias existed in the pooled analysis. When results from two tests contradicted with each other, then resort to the result of Egger’s test for reference.

## Results

### Characteristics of included studies

**Fig. 1:**
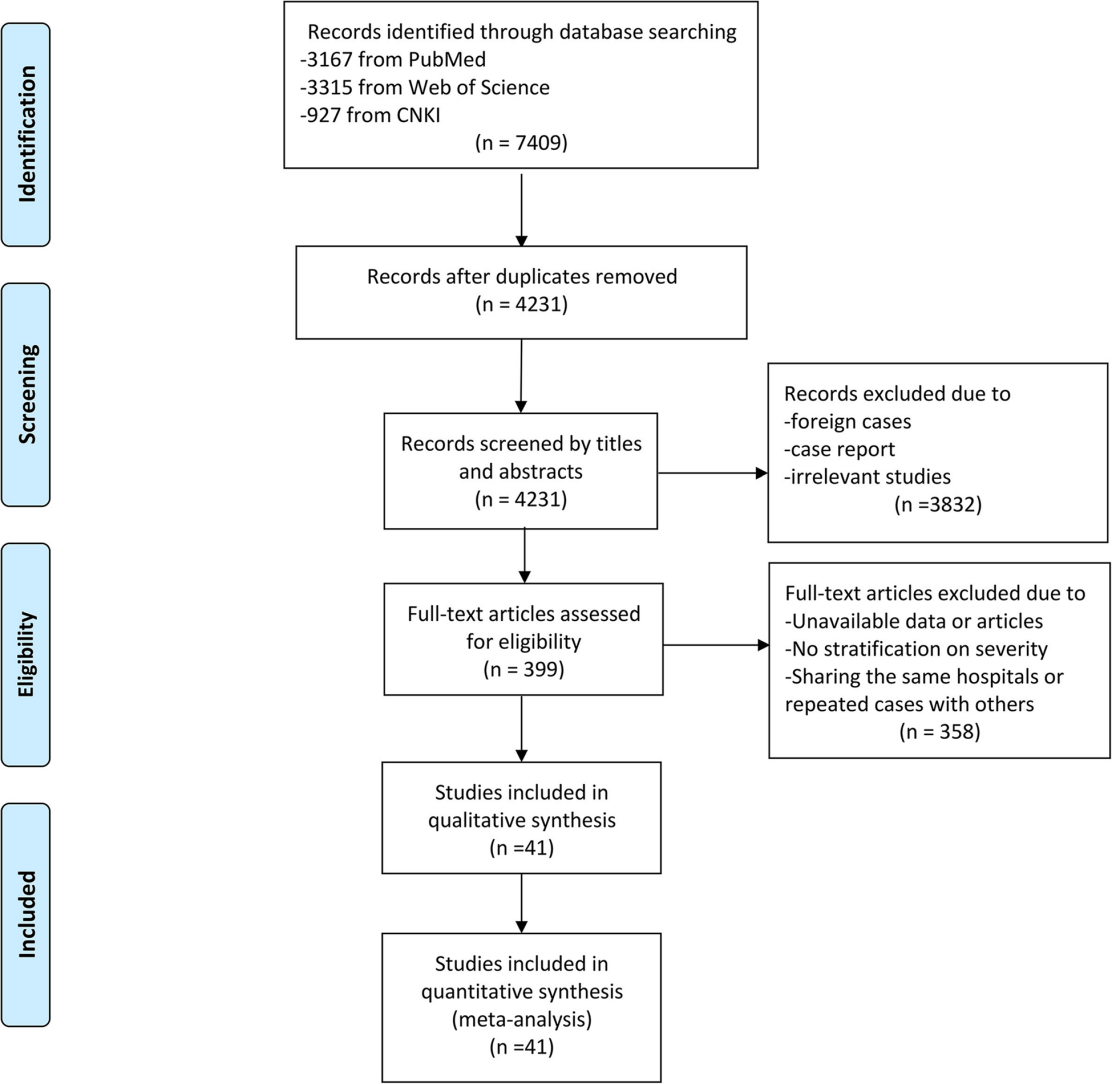
The flow diagram of the number of studies screened and included in the meta-analysis

Initial searching retrieved a total of 7409 citations. After excluding duplicated and irrelevant studies, 399 potentially eligible studies left and were screened for full-text. Finally, we included 41 case series [7–47] for our literature review after excluding 318 records where necessary information was unavailable or no stratification based on severity among patients was displayed and 40 records which shared cases from the same hospitals with other included studies. The flow diagram of the search and study selection process is shown in Fig. 1. The general clinical characteristics of a total of 12,526 COVID-19 patients within 41 included studies are presented in Table 1. All 41 studies were focused on Chinese population and performed at different hospitals in China, among which 18 studies with a total of 6466 cases came from Wuhan and its adjacent cities within Hubei, the origin of COVID-19 outbreak in China, while others were conducted outside Hubei and covered 15 different provinces, including Beijing, Shanghai, Chongqing, Anhui, Hunan, Jiangsu, Jiangxi, Henan, Zhejiang, Guangxi, Guizhou, Guangdong, Liaoning, Jilin and Sichuan. All 41 included studies were further sorted by comorbidities, with 39 on hypertension, 39 on diabetes, 31 on COPD, 30 on cardiovascular diseases, 23 on CLD, 21 on CKD, 20 on malignancy and 16 on cerebrovascular diseases. Fever was the most prevalent symptom and cough the second, followed by myalgia or dyspnea. The age of infected patients was distributed around 50 years old, ranging from infants to very elderly people. The difference of COVID-19 infection between male and female patients was not distinct. As for comorbidity, the proportion of patients with one or more comorbidities in the severe group was noticeably higher than that in the non-severe.

**Table 1:** A baseline characteristic of included studies in COVID-19

	Study	Region	Date	number (n, %)					Age	symptoms (%)				comorbidity (n, %)		
				All	male	female	non-severe	severe		Fever	Cough	Myalgia	Dyspnea	All	non-severe	severe
**1**	**Cheng Kebin**	**Wuhan Jinyintan Hospital, Hubei, China**	**01.01–02.06**	**463**	**244 (53)**	**219 (47)**	**282 (61)**	**181 (39)**	**51 (43,60)**	**90**	**77**	**13**	**42**	**45 (9)**	**25 (10)**	**20 (9)**
**2**	**Fang Xiaowei**	**Anhui Provincial Hospital, Hefei, China**	**01.22–02.18**	**79**	**45 (57)**	**34 (43)**	**55 (70)**	**24 (30)**	**45 ± 16**	**85**	**57**	**34**	**11**	**27 (34)**	**11 (20)**	**16 (66)**
**3**	**Li dan**	**Zhuzhou Central Hospital, Hunan, China**	**01.20–02.27**	**80**	**40 (50)**	**40 (50)**	**63 (79)**	**17 (21)**	**47 (3–90)**	**74**	**71**	**27**	**23**	**22 (27)**	**–**	**–**
**4**	**Li Kunhua**	**The Second Affiliated Hospital of Chongqing Medical University, Chongqing, China**	**01.01–02.29**	**83**	**44 (53)**	**39 (47)**	**58 (70)**	**25 (30)**	**45 ± 12**	**87**	**78**	**18**	**11**	**15 (18)**	**4 (6)**	**11 (44)**
**5**	**Sun Lijun**	**Beijing 302 Hospital, Beijing, China**	**01.20–02.15**	**55**	**31 (56)**	**24 (44)**	**40 (73)**	**15 (27)**	**44 (34–56)**	**82**	**47**	**18**	**9**	**21 (23)**	**9 (15)**	**12 (40)**
**6**	**Xiao Kaihu**	**Chongqing University Three Gorges Hospital, Chongqing, China**	**01.23–02.08**	**143**	**73 (51)**	**70 (49)**	**107 (75)**	**36 (25)**	**45.1 ± 1.0**	**67**	**53**	**26**	**18**	**46 (32)**	**29 (27)**	**17 (47)**
**7**	**Yuan Jing**	**Affiliated Public health hospital of southwest university, Chongqing, China**	**01.24–02.23**	**223**	**105 (47)**	**118 (53)**	**192 (86)**	**31 (14)**	**46.5 ± 16.1**	**53**	**51**	**5**	**5**	**54 (24)**	**40 (21)**	**14 (45)**
**8**	**Zhang Guqin**	**Zhongnan Hospital of Wuhan University, Hubei, China**	**01.02–02.10**	**221**	**108 (49)**	**113 (51)**	**166 (75)**	**55 (25)**	**55 (39–66)**	**91**	**61**	**–**	**29**	**78 (35)**	**40 (24)**	**38 (69)**
**9**	**Zhang Jin-jin**	**No. 7 Hospital of Wuhan, Hubei, China**	**01.10–02.03**	**140**	**69 (49)**	**71 (51)**	**82 (59)**	**58 (41)**	**57 (25–87)**	**92**	**75**	**75**	**37**	**90 (64)**	**44 (54)**	**46 (80)**
**10**	**Zhao Cancan**	**The First Affiliated Hospital of Bengbu Medical College, Anhui, China**	**01.24–02.17**	**189**	**91 (48)**	**98 (52)**	**153 (80)**	**36 (20)**	**46 ± 16**	**97**	**98**	**20**	**11**	**45 (24)**	**25 (16)**	**20 (56)**
**11**	**Zhao Xin-Ying**	**Jingzhou Central Hospital, Hubei, China**	**01.16–02.10**	**91**	**49 (54)**	**42 (46)**	**61 (67)**	**30 (33)**	**46**	**65**	**82**	**17**	**–**	**18 (33)**	**10 (25)**	**8 (53)**
**12**	**Zheng Yongli**	**Chengdu Public Health Clinical Medical Center, Sichuan, China**	**01.16–02.20**	**99**	**51 (51)**	**48 (49)**	**67 (67)**	**32 (33)**	**49 ± 18**	**86**	**85**	**12**	**35**	**41 (41)**	**18 (27)**	**23 (72)**
**13**	**Siqin Zhang**	**Guizhou Provincial Staff Hospital, Guizhou, China**	**02.15–03.31**	**134**	**69 (51)**	**65 (49)**	**115 (86)**	**19 (14)**	**33 (21–46)**	**–**	**–**	**–**	**–**	**25 (18)**	**13 (18)**	**12 (18)**
**14**	**Yiping Wei**	**Suizhou Zengdu Hospital, Hubei, China**	**01.27–03.11**	**276**	**155 (56)**	**121 (44)**	**262 (95)**	**14 (5)**	**51 (41–58)**	**100**	**79**	**9**	**15**	**68 (24)**	**56 (36)**	**12 (9)**
**15**	**Huilin Fang**	**Wuhan Third Hospital, Hubei, China**	**01.27–03.15**	**1280**	**615 (49)**	**655 (51)**	**793 (62)**	**487 (38)**	**63 (51–70)**	**68**	**67**	**6**	**6**	**600 (46)**	**300 (48)**	**300 (45)**
**16**	**Yang Xiuhong**	**Wuhan NO.1 Hospital, Hubei, China**	**02.12–03.07**	**412**	**183 (44)**	**229 (56)**	**111 (27)**	**301 (73)**	**60 ± 14**	**63**	**53**	**23**	**13**	**229 (55)**	**69 (37)**	**160 (69)**
**17**	**Ren Meixin**	**Beijing Youan Hospital, Beijing, China**	**01.21–03.31**	**103**	**45 (44)**	**58 (56)**	**71 (69)**	**32 (31)**	**–**	**–**	**–**	**–**	**–**	**41 (39)**	**19 (42)**	**22 (37)**
**18**	**Wang Ling**	**Huangshi Central Hospital, Hubei, China**	**01.23–03.01**	**92**	**42 (46)**	**50 (54)**	**71 (77)**	**21 (23)**	**50 ± 16**	**70**	**58**	**1**	**–**	**–**	**–**	**–**
**19**	**Yao Chunyong**	**Yicheng People’s Hospital, Hubei, China**	**01.16–02.09**	**66**	**–**	**–**	**55 (83)**	**11 (17)**	**21–71**	**85**	**71**	**–**	**–**	**–**	**–**	**–**
**20**	**Lv Yaodong**	**Nanning Fourth People’s Hospital, Guangxi, China**	**01.23–03.02**	**58**	**29 (50)**	**29 (50)**	**44 (76)**	**14 (24)**	**55 (3 m-90 yr)**	**50**	**57**	**36**	**17**	**26 (44)**	**15 (51)**	**11 (37)**
**21**	**Cheng Fang**	**Wenzhou Central Hospital, Zhejiang, China**	**01.15–02.29**	**77**	**43 (56)**	**34 (44)**	**50 (65)**	**27 (35)**	**46 ± 16**	**90**	**70**	**8**	**30**	**–**	**–**	**–**
**22**	**Shan-Yan Zhang**	**Multiple hospitals in ZheJiang Province, China**	**01.17–02.12**	**788**	**407 (52)**	**381 (48)**	**710 (90)**	**78 (10)**	**–**	**81**	**64**	**12**	**–**	**218 (27)**	**171 (42)**	**47 (12)**
**23**	**Zhengtong Lv**	**Jiangan Fangcang shelter hospital, Hubei, China**	**02.01–03.31**	**409**	**188 (46)**	**221 (54)**	**361 (88)**	**48 (12)**	**50 ± 12**	**96**	**74**	**16**	**–**	**–**	**–**	**–**
**24**	**Zhichao Feng**	**The whole Hunan province, China**	**01.17–02.28**	**564**	**284 (50)**	**280 (50)**	**495 (88)**	**69 (12)**	**47 (36, 58)**	**63**	**57**	**4**	**5**	**132 (23)**	**95 (33)**	**37 (13)**
**25**	**Chen Zhao**	**Union Hospital, Tongji Medical College, Huazhong University of Science and Technology, Wuhan, Hubei, China**	**02.13–02.25**	**172**	**82 (48)**	**90 (52)**	**112 (65)**	**60 (35)**	**65 (57, 71)**	**63**	**55**	**22**	**–**	**95 (55)**	**57 (69)**	**38 (42)**
**26**	**Lingshuang Sheng**	**Tongji Hospital of Tongji Medical College of Huazhong University of Science and Technology, Wuhan, Hubei, China**	**–**	**232**	**–**	**–**	**102 (44)**	**130 (56)**	**68 (58–90)**	**–**	**–**	**–**	**–**	**133 (57)**	**–**	**–**
**27**	**Yifei Nie**	**279 hospitals in Henan Province, Henan, China**	**01.23–02.05**	**655**	**367 (56)**	**288 (44)**	**583 (90)**	**72 (10)**	**43 ± 15**	**–**	**–**	**–**	**–**	**–**	**–**	**–**
**28**	**Sheng-long Chen**	**32 hospitals in Guangdong Province, Guangdong, China**	**01.14–03.16**	**1168**	**560 (48)**	**608 (52)**	**1020 (87)**	**148 (13)**	**43 (32–57)**	**69**	**49**	**8**	**7**	**245 (20)**	**172 (30)**	**73 (12)**
**29**	**B. Cheng**	**The Central Hospital of Wuhan, Tongji Medical College, Huazhong University of Science and Technology, Wuhan, Hubei, China**	**01.01–03.20**	**456**	**211 (46)**	**245 (54)**	**205 (45)**	**251 (55)**	**54 ± 18**	**65**	**53**	**34**	**22**	**–**	**–**	**–**
**30**	**Songqiao Liu**	**24 hospitals in Jiangsu province, China**	**01.10–03.15**	**625**	**329 (53)**	**296 (47)**	**561 (90)**	**64 (10)**	**44 ± 17**	**66**	**55**	**–**	**–**	**–**	**–**	**–**
**31**	**XiaoYu**	**The Center for disease control and prevention in ShangHai, China**	**01.24–02.19**	**333**	**161 (48)**	**172 (52)**	**307 (92)**	**26 (8)**	**50 (35, 63)**	**82**	**41**	**8**	**1**	**107 (32)**	**91 (56)**	**16 (9)**
**32**	**Yanpei Zhang**	**the People’s Hospital of Honghu, Hubei, China & the First Affiliated Hospital of Nanchang University, Jiangxi, China**	**01.01–03.18**	**365**	**176 (48)**	**189 (52)**	**339 (93)**	**26 (7)**	**46 ± 15**	**61**	**53**	**0**	**4**	**–**	**–**	**–**
**33**	**Yuan Cen**	**Huoshenshan Hospital & General Hospital of the central theater Command of the people’s Liberation Army & Mobile cabin hospitals in Wuhan, Hubei, China**	**–**	**1007**	**493 (49)**	**514 (51)**	**720 (71)**	**287 (29)**	**61 (49–68)**	**75**	**65**	**2**	**36**	**364 (36)**	**195 (39)**	**169 (32)**
**34**	**Jixiang Zhang**	**Renmin Hospital of Wuhan University, Hubei, China**	**01.11–02.06**	**663**	**321 (48)**	**342 (52)**	**254 (39)**	**409 (61)**	**55 (44, 69)**	**79**	**62**	**10**	**24**	**–**	**–**	**–**
**35**	**Qing Zhang**	**First Hospital of Jilin University and Infectious Diseases Hospital in Changchun City, Jilin, China**	**01.23–02.25**	**41**	**23 (56)**	**18 (44)**	**33 (80)**	**8 (19)**	**45 (31, 53)**	**78**	**71**	**7**	**20**	**–**	**–**	**–**
**36**	**Jing-Bo Wang**	**Sixth People’s Hospital of Shenyang, Liaoning, China**	**01.24–02.17**	**56**	**24 (43)**	**32 (57)**	**45 (80)**	**11 (20)**	**45 (21–80)**	**75**	**61**	**11**	**27**	**26 (46)**	**18 (75)**	**8 (25)**
**37**	**Nannan Zhang**	**Jining Infectious Disease Hospital, Jining City, Shandong, China**	**01.24–03.01**	**78**	**60 (77)**	**18 (23)**	**72 (92)**	**6 (8)**	**43 ± 15**	**81**	**46**	**5**	**10**	**–**	**–**	**–**
**38**	**Qingchun Yao**	**Dabieshan Medical Center, Huanggang city, Hubei, China**	**01.30–03.11**	**108**	**43 (40)**	**65 (60)**	**83 (77)**	**25 (23)**	**52 (37–58)**	**74**	**78**	**26**	**14**	**25 (23)**	**12 (27)**	**13 (20)**
**39**	**Zixin Shu**	**Hubei Provincial Hospital of Traditional Chinese Medicine, Hubei, China**	**01.15–03.02**	**293**	**135 (46)**	**158 (54)**	**207 (71)**	**86 (29)**	**57 ± 15**	**35**	**51**	**–**	**–**	**178 (60)**	**108 (80)**	**70 (44)**
**40**	**Huihuang Huang**	**Fifth Medical Center of Chinese PLA General Hospital, Beijing, China**	**01.13–03.10**	**64**	**37 (58)**	**27 (42)**	**43 (67)**	**21 (33)**	**47 ± 18**	**–**	**–**	**–**	**–**	**–**	**–**	**–**
**41**	**Chang-Zheng Wang**	**Xiangyang No.1 People’s Hospital, Hubei University of Medicine, Xiangyang, Hubei, China**	**01.10–02.28**	**85**	**45 (53)**	**40 (47)**	**39 (46)**	**46 (54)**	**59 ± 15**	**89**	**76**	**42**	**9**	**–**	**–**	**–**

Age presented in the form of median with range (−) or interquartile range (,), or mean with SD (±) according to the original data

### Comorbidities in COVID-19 cases

**Fig. 2:**
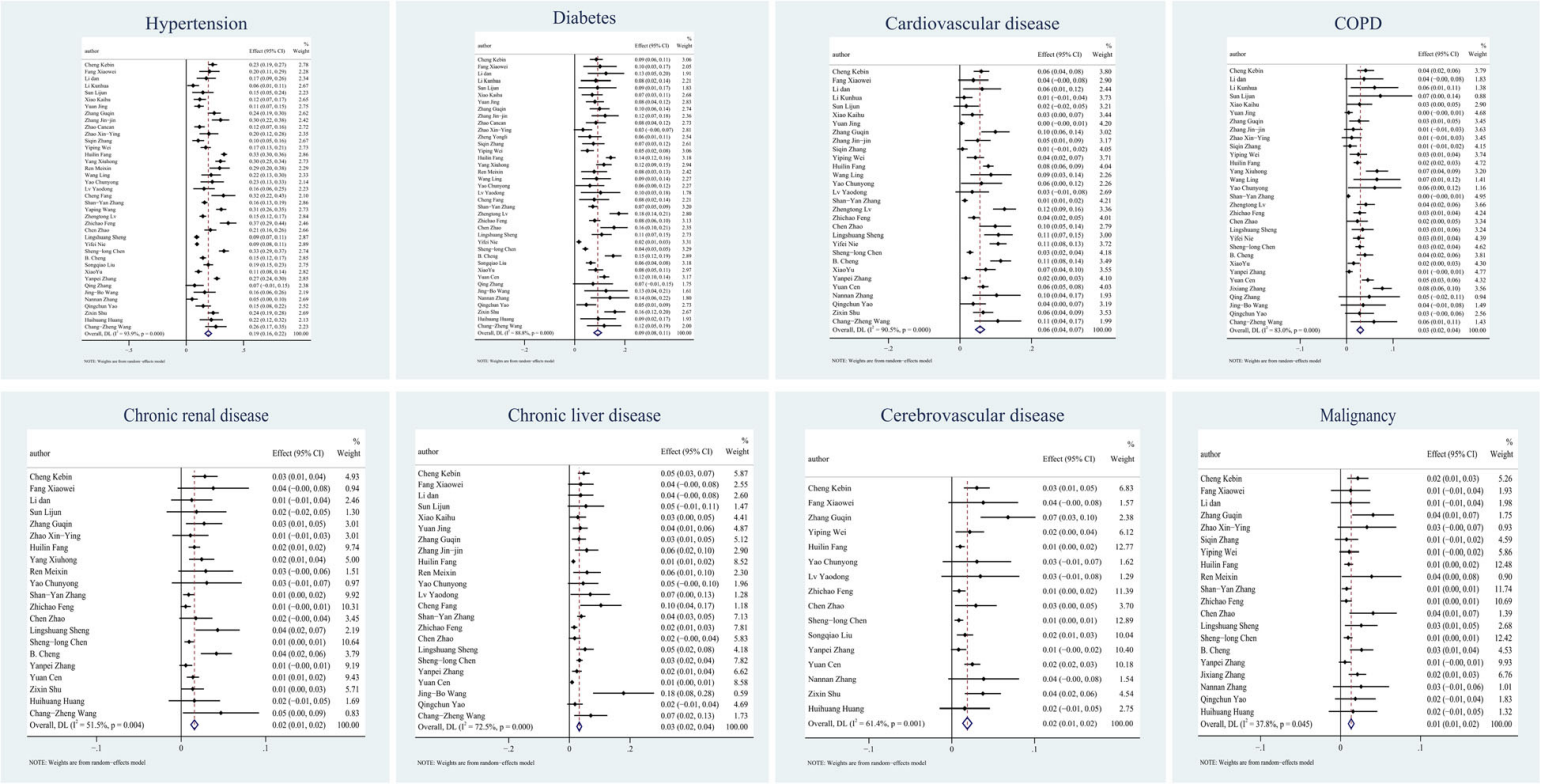
The Meta-analysis of the prevalence of comorbidities in COVID-19 cases. Weight was calculated from the binary random-effects model analysis. The value represents the proportion of 8 diseases and 95% confidence intervals, respectively, together with heterogeneity analysis carried by Q-test and I^2^ index. Parts for each comorbidity are arrayed in the figure orderly as follows: hypertension, diabetes, cardiovascular disease, COPD, chronic kidney disease, chronic liver disease, cerebrovascular disease, malignancy

Pooled estimation of prevalence of different comorbidities among COVID-19 patients are provided in Fig. 2 as forest plots and summarized in Table 2. The results showed that the comorbidities successively arrayed by proportion were hypertension (19, 95% CI 16–22%), diabetes (9, 95% CI 8–11%), cardiovascular diseases (6, 95% CI 4–7%), CLD (3, 95% CI 2–4%), COPD (3, 95% CI 2–4%), cerebrovascular diseases (2, 95% CI 1–2%), CKD (2, 95% CI 1–2%) and malignancy (1, 95% CI 1–2%). Based on Cochrane’s Q test, significant heterogeneity was observed in the estimates of all comorbidities, with I^2^ ranging from 37.8 to 93.9%. Results of u-test was listed in Table 3, the proportion of malignancy and cardiovascular and cerebrovascular diseases in COVID-19 patients was much higher than that in the general population [48, 49] (***, *P* < 0.001), while the prevalence of hypertension, diabetes, COPD, CLD and CKD were lower than that of the overall Chinese population [50–53] (***, *P* < 0.001).

**Table 2:** The Meta-analysis of the prevalence of comorbidities in COVID-19 cases

Diseases	Pooled prevalence	95% CI	I^2^	***P***
Hypertension	0.19	0.16–0.22	93.9	< 0.001
Cardiovascular disease	0.06	0.04–0.07	90.5	< 0.001
Diabetes	0.09	0.08–0.11	88.8	< 0.001
COPD	0.03	0.02–0.04	83.0	< 0.001
Cerebrovascular disease	0.03	0.02–0.05	61.4	0.001
Chronic renal disease	0.02	0.01–0.02	51.5	0.004
Chronic liver diseases	0.03	0.02–0.04	72.5	< 0.001
Malignancy	0.01	0.01–0.02	37.8	0.045

Pooled prevalence and 95% confidence intervals (95% CI) of 8 diseases were presented respectively, together with the result of heterogeneity analysis described as I^2^ and *P* value. *P* value for heterogeneity among studies assessed with Cochran’s Q test

**Table 3:** The comparison of prevalence of 8 comorbidities between included COVID-19 patients and the Chinese population [48–53]

Disease	Prevalence from Meta-analysis (%)	Prevalence from the general population (%)	*P*-value ^***^	Reference ^a^
Hypertension	19	23.2	< 0.001	2018 [48]
Cardiovascular disease	6	1.8	< 0.001	2018 [48]
Cerebrovascular disease	2	0.9	< 0.001	2018 [48]
Diabetes	9	10.9	< 0.001	2013 [50]
COPD	3	8.6	< 0.001	2018 [51]
Chronic liver disease	3	> 24.8	< 0.001	2019 [52]
Chronic kidney disease	2	9.5	< 0.001	2017 [53]
Malignancy	1	0.6	< 0.001	Recent 5-year [49]

*** P < 0.001 The significant difference between the two groups is indicated by the u-test

^*a*a^References for the incidence of the indicated diseases in the general population were included in parenthesis. Dates under the heading of Reference refer to the year in which statistic data was collected before

**Fig. 3:**
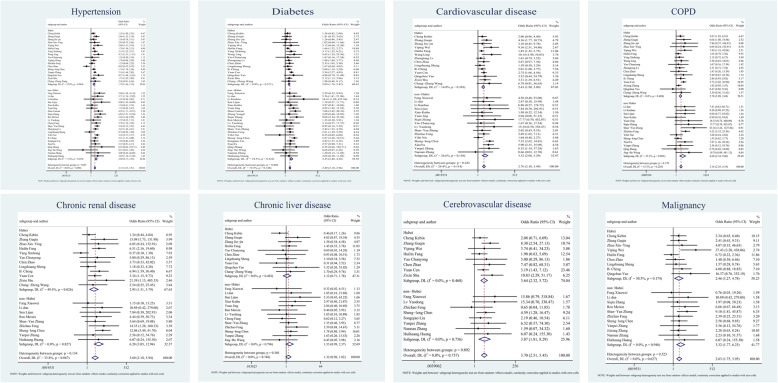
The risk of comorbidities in severe patients compared to Non-severe patients and subgroup analysis. Forest plots illustrate the comparison of the specific comorbidity’s prevalence in severe and non-severe patients, together with the heterogeneity analysis carried by the Q-test and I^2^ index. In these panels, the size of the diamonds reflects the sample size, and the whiskers extend to the lower and upper values of the 95% confidence interval (CI). Parts for each comorbidity are arrayed in the figure orderly as follows: hypertension, diabetes, cardiovascular disease, COPD, chronic kidney disease, chronic liver disease, cerebrovascular disease, malignancy.

Evaluation for the impact of these comorbidities on COVID-19 severity was presented as forest plots in Fig. 3, showing that the proportion of all comorbidities in severe patients was significantly higher than that in non-severe patients, with the exception of CLD (OR = 1.32, 95% CI 0.96–1.82). Cerebrovascular disease had the highest OR value of 3.70 (95% CI 2.51–5.45), sequentially followed by CKD (OR = 3.60, 95% CI 2.18–5.94), COPD (OR = 3.14, 95% CI 2.35–4.19), cardiovascular disease (OR = 2.76, 95% CI 2.18–3.49), diabetes (OR = 2.49, 95% CI 2.10–2.96), malignancy (OR = 2.63, 95% CI 1.75–3.95) and hypertension (OR = 2.13, 95% CI 1.81–2.51). According to Cochran’s Q test result, apart from hypertension which had the *P*-value of 0.007 and CKD which had the P-value of 0.067 and thus was heterogeneous, no obvious heterogeneity (I^2^ ranged from 0 to 24.4%) existed among other comorbidities.

The *P* values of Egger’s and Begg’s tests towards each comorbidity were above or approximate to 0.05, which appears to have little publication bias presented in each result. The detailed results of the tests for publication bias were summarized in Table 4 and the result interface from Stata15 can be found in the additional file [see Additional file 2]. Subgroup analysis based on patient source was conducted and presented within Fig. 3 as forest plots and the results were summarized in Table 5. In all comorbidities, the OR value of non-Hubei group was higher than that of Hubei group, and even doubled in some diseases, such as CKD (6.28 vs 2.95) and hypertension (3.36 vs 1.45).

**Table 4:** The results of publication bias assessment by Egger’s test and Begg’s test

Comorbidities	Egger’s test (P)	Begg’s test (P)
Hypertension	0.375	0.578
Diabetes	0.570	0.828
Cardiovascular diseases	0.115	0.286
COPD	0.144	0.185
Malignancy	0.050	0.048
Chronic liver diseases	0.798	0.673
Chronic kidney diseases	0.260	0.566
Cerebrovascular diseases	0.372	0.620

**Table 5:** The results of subgroup analysis

Comorbidities	Hubei OR (95%CI)	non-Hubei OR (95%CI)	total OR (95%CI)
Hypertension	1.79 (1.46–2.19)	2.96 (2.17–3.35)	2.13 (1.81–2.51)
Diabetes	2.09 (1.76–2.47)	3.25 (2.48–4.26)	2.49 (2.10–2.96)
Cardiovascular diseases	2.43 (1.90–3.09)	3.32 (2.08–5.29)	2.76 (2.18–3.49)
COPD	2.78 (2.09–3.69)	4.60 (2.34–9.06)	3.14 (2.35–4.19)
Malignancy	2.46 (1.27–4.78)	3.32 (1.77–6.23)	2.63 (1.75–3.95)
Chronic liver diseases	1.12 (0.71–1.78)	1.53 (0.99–2.37)	1.32 (0.96–1.82)
Chronic kidney diseases	2.95 (1.51–5.79)	6.28 (3.05–12.94)	3.60 (2.18–5.94)
Cerebrovascular diseases	3.64 (2.32–5.72)	3.87 (1.81–8.29)	3.70 (2.51–5.45)

## Discussion

Compared with previous reports, the present systematic review and meta-analysis contained a relatively large sample of more than 12,000 cases covering 16 provinces in China and collected the most recent data (till January 2021) comparing subgroups of Hubei and non-Hubei to show the prevalence of 8 comorbidities and their impact on developing severity of COVID-19. On the whole, our results were in the line with previous studies [54–56]. Therefore, it is safe to draw that hypertension, diabetes, cardiovascular diseases are the most prevalent concomitant diseases in patients with COVID-19 [57].

When compared with the prevalence of these indicated morbidities in the general Chinese population, the pooled prevalence of cardio-cerebrovascular diseases and cancer were obviously higher in the hospitalized COVID-19 patients; on the contrary, that of COPD, CKD, and CLD were remarkably lower. Additionally, the estimated prevalence of hypertension and diabetes in the COVID-19 patients was not evidently different from that of the overall population, suggesting that such patients may not be more susceptible to SARS-CoV-2 infection. (Details see Table 3).

We have compared 4 recent meta-analyses [54, 55] [58, 59] on the correlation of various comorbidities with disease progression and all of them agreed that hypertension, cardiovascular disease, diabetes, and COPD are commonly associated with the poor outcomes in COVID-19. Three investigations have shown a significant association of CKD with severe COVID 19 cases [54, 55] [58]. According to a large multinational meta-analysis, CLD and CKD were predictors for severe COVID-19 with a similar power [55]. Besides, Singh A.K. et al. [54] reported that cancer (RR = 2.48, 95% CI 1.46–4.19) was significantly associated with a higher risk of severe COVID-19, compared to patients without comorbidities. However, Bolin Wang et al. [59] did not find the correlation between the above 3 comorbidities and aggravation of COVID-19. Few meta-analyses considered cerebrovascular disease independently, and some incorporated it with cardiovascular disease or overlooked it for a small sample size. Bolin Wang et al. [59] pointed that cerebrovascular disease was an important independent risk factor for COVID-19 (OR = 3.89), which is similar to the present meta-analysis. On the contrary, Singh A.K. et al. [54] indicated that cerebrovascular disease was not significantly associated with severe COVID-19 (RR = 1.73 95% CI 0.74–4.05).

The present study revealed that with the exception of CLD, other 7 underlying diseases exhibited statistically significant correlation with severe COVID-19, of which cerebrovascular disease was the strongest risk factor, followed by CKD and COPD. Our results are in general in line with previous reports although variations and even discrepancy exist in some of individual studies. This may be due to differences in size and sources of the sample included, different statistical methods used, outcome criteria, and ethnic background.

According to the estimated prevalence and the odds risk of developing severe outcomes, 8 comorbidities can be stratified into 3 groups. Firstly, pre-existing cardio-cerebrovascular diseases and malignancy may both impose higher susceptibility to SARS-CoV-2 infection and develop to severe cases, which alerts both intensive health care and such patients themselves during the outbreak. The prevalence of cardia-cerebrovascular diseases in patients with COVID-19 was much higher than that of general population, which may be explained by the long-term use of angiotensin-converting enzyme inhibitors (ACEI) or angiotensin receptor blockers (ARBs), which upregulate ACE2, leading to an increase in contracting SARS-CoV-2 [60, 61].

In keeping with Li et al. [62], our results showed that patients with hypertension, cardio-cerebrovascular diseases, and diabetes had more than 2-fold, 3-fold and 2-fold increased risk of developing severe COVID-19, respectively. These diseases share some common features, including vascular endothelial injury, dysfunctional hemostatic system, and pro-inflammatory state or chronic inflammation [63]. These conditions usually appear with the overload of cytokines induced by viral infection and are conducive to cytokine storms, leading to critical illness [64]. According to pathological analysis, most of the deaths are attributed to cytokine storm-triggered multiple Organ Failure (MOF) and Acute Respiratory Distress Syndrome (ARDS) [65]. On the other hand, COVID-19 patients with concurrent cardiovascular diseases such as arrhythmia and atherosclerosis can increase the likelihood of thromboembolic events, which may further contribute to fatal cerebral ischemia and acute stroke [60, 66]. And this trend could be accelerated by pre-existing compromised cerebral vasculature, hypercoagulability, and vessel inflammation in patients with cerebrovascular diseases. Other critical complications induced by the hypercoagulable state included systemic sepsis and micro-thrombosis formation in pulmonary blood vessels, which implies the possibility of life-threatening pulmonary embolism [60]. Although the pathophysiology behind may be overlapped, it is worth noting that the risk of developing severe outcomes where cerebrovascular and cardiovascular diseases pose on COVID-19 patients are mutually independent, and seem not affected by the presence of hypertension or diabetes [60].

Secondly, the overall pooled prevalence of cancer in COVID-19 infections was higher as compared to the overall population in China (0.6%), ranging from 1 to 3.9% [54, 56] [67, 68], within which our results fall (1, 95% CI 1–2%). This may be associated with the suppressed immunity that diminishes the ability of host surveillance. In addition, frequent hospital visits or hospitalization of cancer patients might increase the risk of contracting SARS-CoV-2. The study of Liang et al. [67] reported that patients with cancer had a higher risk of severity than non-cancer patients, possibly due to suppressed immune response as a result of chemo- and radio-therapies.

Thirdly, patients with COPD and CKD appear to have low risk of contracting SARS-CoV2 but tend to become severe once infected. Given the prominent pulmonary manifestations in COVID-19 patients and abundant ACE2 in the lung, it is reasonable to conceive severe outcomes of COVID-19 with the underlying COPD. However, published and this meta-analysis revealed the low prevalence of COPD in COVID-19 patients [69, 70]. One plausible explanation is that the long-term use of bronchodilators for the COPD patients, such as inhaled steroids, beta-agonists, or anticholinergics [71], could inhibit viral replication partly by lowering coronavirus receptor expression, suppressing the function of acidic endosomes, and modulating inflammation induced by infection in the airway, thus reducing the susceptibility to infection [72]. However, the patients with COPD are more vulnerable to the development of severe and critical conditions. A gene profile study has recently demonstrated that ACE-2 expression is significantly elevated on bronchial epithelial cells in the COPD patients, as compared to control subjects, which might contribute to exacerbated progression [73]. Additionally, it has been documented that COPD can lead to systemic hypoxia, which is sensitive to the induction of cytokine storms [74].

We did not find significant correlation between CLD and the risk of COVID-19 infection. It is well-known that there is a considerable burden of CLD (> 24.8%) in Chinese population. The reason for the low prevalence of CLD in Chinese COVID-19 patients is unclear. Two independent multi-national meta-analyses with 38,000 [55] and 24,299 [75] cases showed that that worldwide patients with CLD were more inclined to develop severe outcomes of COVID-19 than those without.

## Conclusion

Currently, the reliability of results from most clinical studies is limited by small sample size and isolated source of cases. Few systemic reviews shed light on the comprehensive perspective of various underlying diseases. In this meta-analysis, we analyzed eight different common comorbidities about their prevalence among COVID-19 patients and found that COPD, cardio-cerebrovascular diseases, diabetes, hypertension, and CKD were significant risk factors in development to severe and critical cases. As the global population with comorbidity is rapidly growing and respiratory infections like COVID-19 seriously threaten public health, our results alert both patients and medical workers about the impact of various comorbidities on SARS-CoV-2 infection and deterioration of pneumonia and highlight the necessity of tailored therapy and monitoring those with basic diseases to avoid further cost and deaths.

## Supplementary Information

**Additional file 1.** Study design and performance according to the Preferred Reporting Items for Systematic Reviews and Meta-Analyses (PRISMA) guidelines.

**Additional file 2.** Primary data extracted from included studies and sorted based on different comorbidities, computational process of u-test and the result pages of begg’s test and egger’s test in Stata15.

## Data Availability

All data generated or analyzed during this study are included in this manuscript and its supplementary information files.

## Abbreviations

COVID-19: Coronavirus disease 2019
SARS-CoV-2: Severe acute respiratory syndrome coronavirus-2
MERS: Middle East Respiratory Syndrome
COPD: Chronic obstructive pulmonary disease
CLD: Chronic liver disease
CKD: Chronic kidney disease
MOF: Multiple organ failure
ARDS: Acute respiratory distress syndrome
ACEI: Angiotensin-converting enzyme inhibitors
ARBs: Angiotensin receptor blockers
